# Hybrid high-concentration photovoltaic system designed for different weather conditions

**DOI:** 10.1038/s41598-023-32128-z

**Published:** 2023-03-30

**Authors:** Chi Sun, Chi-Shou Wu, Yong-Sheng Lin, Tsung Sheng Kao, Shuo-Ting Fang, Yao-Hsuan Chiu, Ching-Cherng Sun

**Affiliations:** 1 Department of Photonics, College of Electrical and Computer Engineering, National Yang Ming Chiao Tung University, Hsinchu, 30010 Taiwan; 2 Department of Optics and Photonics, National Central University, Jhongli, Taoyuan, 32001 Taiwan; 3 Department of Electrophysics, National Yang Ming Chiao Tung University, Hsinchu, 30010 Taiwan

**Keywords:** Solar energy and photovoltaic technology, Imaging and sensing

## Abstract

In this study, we propose a novel high-concentration photovoltaic (HCPV) cell by considering both the light leakage characteristics of the Fresnel-lens-based solar cell modules and the performance issues arising from cloud shading in practical use. We use our self-constructed systems to conduct field measurements for up to half a year under various environmental conditions. According to the acquired results, it was surprising to know that in the area other than the focusing area, the so-called light leakage region, there always bears illuminance of about 20,000–40,000 lx whether it is a sunny day or a cloudy day with different cloud conditions. Such an interesting result is caused by the light scattering of the clouds and the inherent leakage characteristic of a Fresnel lens. To prove this important finding, we simulated the illuminance of the Fresnel lens structure used in the measurement with apertures of different sizes to determine the detected area. In the laboratory, the diffuse plates were used to mimic the situation of varying cloud layer thicknesses. The trend of calculated and measured results fitted well with the field measurements. Also, the experimental and simulation results show that the round angle and draft facet of the Fresnel lens were responsible for light leakage. This finding prompted us to propose a hybrid high-concentration solar module in which more cost-effective polycrystalline silicon solar cells are placed around the high-efficiency wafer of HCPV to capture the dissipated light leakage and convert it into usable electricity.

## Introduction

Among the 17 interlinked global sustainable development goals (SDGs) set by the United Nations General Assembly in 2015, affordable and clean energy is an essential indicator of a better, more sustainable future for all. Therefore, for clean and sustainable energy generation, researchers use various natural resources to obtain so-called green energy, including wind energy, geothermal energy, hydroelectricity, tidal power, solar energy, etc. Among these, solar photovoltaics are less limited by terrain or location and are an energy source that can be obtained almost anywhere in the world^1^. Therefore, effectively converting the light energy from the sun into electrical energy that can be easily used has always been the ultimate goal of the development of human life. Solar cell panels made from semiconductor materials are recognized as the most economical manufactured solar power generation device. Over the past few decades, this device has become widely exploited to generate electricity from the sun rays. They can be found on the roofs of public buildings and individual homes worldwide. Generally, the materials used in semiconductor-based solar panels are polycrystalline silicon or III–V and II–VI compound semiconductors, while their photo conversion efficiencies are 15% and 45%, respectively^2–4^. Regarding the product price, the cost of solar panels made with III-V or II-VI compound semiconductors is much higher than that of polysilicon solar panels. However, for efficiency considerations, III-V or II-VI semiconductors are still used for solar panels in solar power plants. They are used in accompanying sun-tracking systems to improve power generation efficiency^5^. Moreover, we can configure an optical lens above the compound semiconductor-based solar panel to effectively focus the nearly parallel incident sunlight on a limited area of such high-cost solar cells^6,7^. Solar cell modules with this configuration are called high-concentration photovoltaic (HCPV)^8,9^, meaning that through the design and use of optical elements, most of the solar energy can be collected on a small-size solar wafer. In this way, not only can high-performance compound semiconductor materials be fully utilized for solar cell power supply, but also the cost can be greatly reduced due to the reduction of materials used.

In the HCPV systems, the direction of sunlight on the lens is closely related to the amount of solar energy that can be collected by the solar cell, so we need to use a time-varying sun-tracking system to obtain the highest conversion efficiency. Therefore, the structure of the focusing lens also requires a particular design. In addition to the proper focusing distance, we aim to increase the number of focusing lenses per unit area and reduce the weight of the lens itself as much as possible. Conventional focusing lenses are generally challenging to design because of the acceptable focal length and the corresponding lens size, making it difficult to achieve these optimal requirements simultaneously, which in turn makes the price of the sun-chasing system higher. An effective way to solve this problem is to use a Fresnel lens^10^. Xie and Sierra et al. studied the application of the Fresnel lens in high solar energy concentration^11,12^. Chen and Yamada et al. proposed the Fresnel lens design to improve the distribution of illuminance uniformity^13,14^, but they didn’t consider the issue of light leakage of the Fresnel lens and power generation efficiency under heavy-cloud weather conditions. Although the HCPV system combined with the Fresnel lens effectively utilizes the energy of sunlight, as the clouds in the sky change, the Fresnel lens cannot effectively gather the rays from the sun, resulting in a decrease in HCPV power generation efficiency. This paper presents a novel discovery of light leakage in an HCPV, that could maintain a certain level of illuminance under different cloud conditions, and proposes a hybrid light collection system appropriate to different weather conditions, and the power generation efficiency stays optimal. New technical findings will be demonstrated through field measurement under various cloud conditions.

## The light leakage by the Fresnel lens

**Figures 1:**
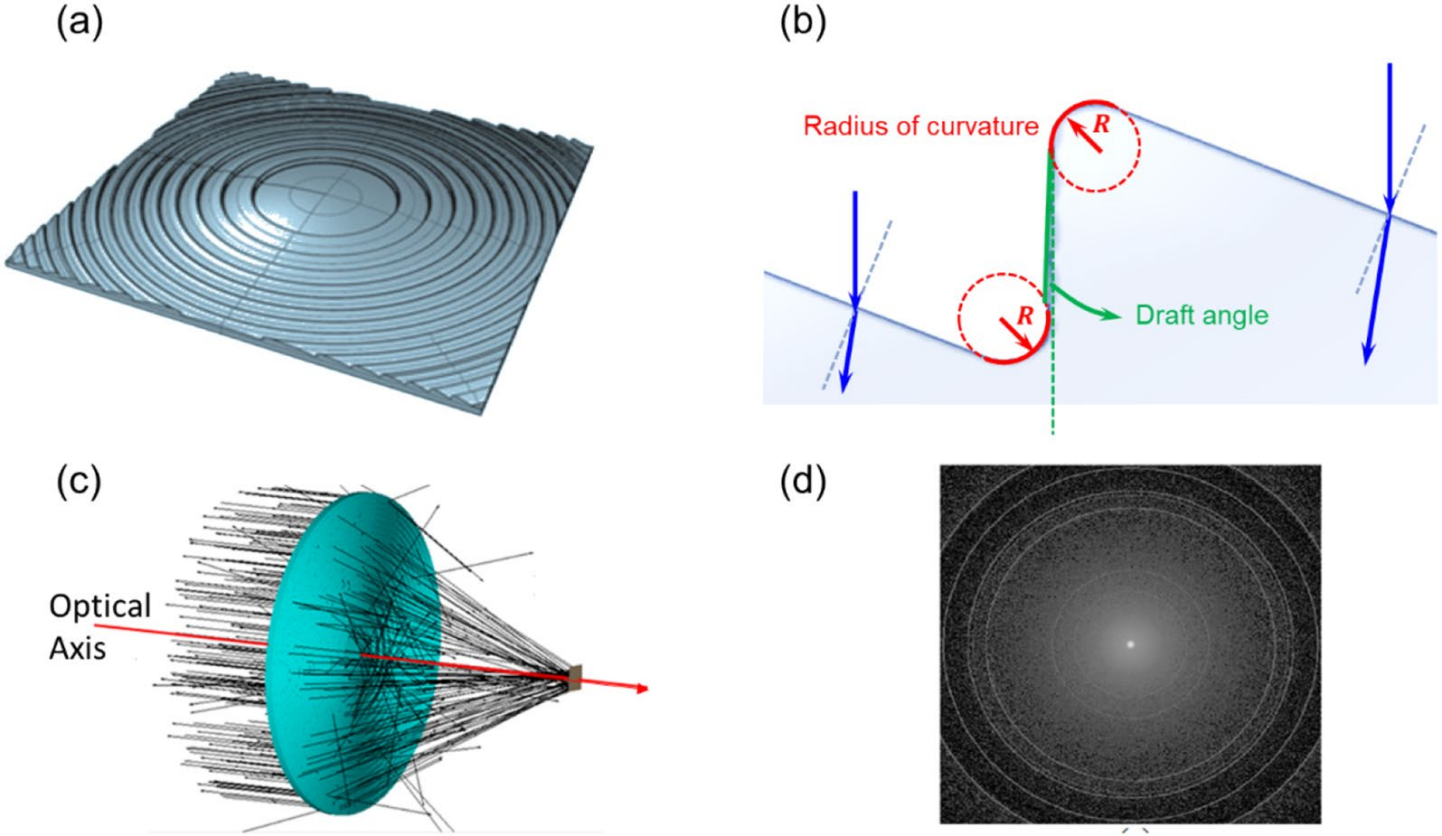
Schematic diagram of the Fresnel lens structure and the simulated beam propagation. (**a**) The structure of the Fresnel lens. (**b**) The diagrammatic sketch represents the zoom-in image of the grooves in Fresnel lenses. (**c**) Ray-tracing simulation and a set of ray fans of the Fresnel lens. (**d**) The simulated optical flux distribution across the focal plane. The illuminance shown outside the focusing area came from the leakage light.

A Fresnel lens is a type of composite compact lens, and the structural design is schematically shown in Fig. 1a, allowing the construction of lenses of large aperture and short focal length without the mass and volume of material that would be required by a conventional lens design^15–17^. Therefore, a Fresnel lens can be made much thinner than a conventional lens. Fresnel lenses are designed to significantly reduce the weight of the lens so that it can meet the requirements of a sun-tracking system. However, unlike conventional lenses, non-smooth and non-continuous lens surfaces distributed in multiple segments may cause inevitable significant light leakage due to manufacturing errors such as the radii of curvature and draft angles of the fabricated Fresnel lenses, as indicated in Fig. 1b. Subtle changes in these structures may make part of the parallel incident sunlight unable to be effectively focused into the energy harvesting range in the solar cell module through the Fresnel lens, resulting in decreased power generation efficiency. We used a Fresnel lens for simulation calculation with Advanced System Analysis Program (ASAP)^18^, where the radius of curvature of the round angles (RVRA) and draft angles were around 0.1 mm and 1°, respectively. The width of the Fresnel lens was 129 mm and the thickness is 1.81 mm. The simulation calculation results are presented in Fig. 1c by Monte Carlo ray tracing^19^, where the total simulation ray number was 10,000,000. When using the Fresnel lens as the focusing lens of HCPV, although most of the incident beams can be effectively concentrated into the focus area, which may be equivalent to the energy collection area, still part of the light energy is dissipated out of the energy collection region. The size of the focusing area in the simulation was about 1.1 mm × 1.1 mm, the power ratio of focus was 63%, and the light leakage ratio outside the focusing area was 37%.

Another practical problem encountered when using HCPV solar modules is that the sky cannot always be clear and cloudless. So, when a cloud layer passes through the sunlight and the solar modules, the sun rays are multiply scattered by the water molecules inside the cloud, causing the change of the traveling direction of the light entering the solar module, which was nearly parallel. The change in the direction of sunlight travel is related to the thickness of the clouds. When the cloud thickness is thin, most of the sunlight's forward direction is not affected, so the sunlight can still be concentrated on the solar cell modules. However, when the thickness of the cloud layer increases to a certain thickness, the sun rays passing through the cloud layer collide with water molecules randomly, resulting in a random scattering path. Thus the sunlight is no longer collimated when it reaches the Fresnel lens, and therefore cannot be efficiently concentrated on the solar cell with a lens, and results in a decrease of illuminance on the solar cell. In this case, if an III-V semiconductor-based solar cell is used, the HCPV has almost no power conversion efficiency due to its small light-harvesting area.

## Characterization of Fresnel lenses in light leakage

To effectively obtain the dissipated light energy which is due to the use of Fresnel lenses and make high-efficiency solar cell modules that can be used in various weather conditions, this research work first calculated and measured the light energy in the light leakage area of Fresnel lenses with different structures under monochromatic light irradiation. Next, we used diffuse plates with varying penetration rates to mimic the outdoor conditions of different cloud layer thicknesses and measured the lateral light intensity distribution on the solar cell module. Finally, summarizing the results of outdoor field measurements, we propose a hybrid solar high-concentration photovoltaic module, expecting that such a system can combine the advantages of HCPV and polycrystalline-silicon-based solar panels simultaneously and achieve comparable power conversion efficiency under different weather conditions.

**Figure 2:**
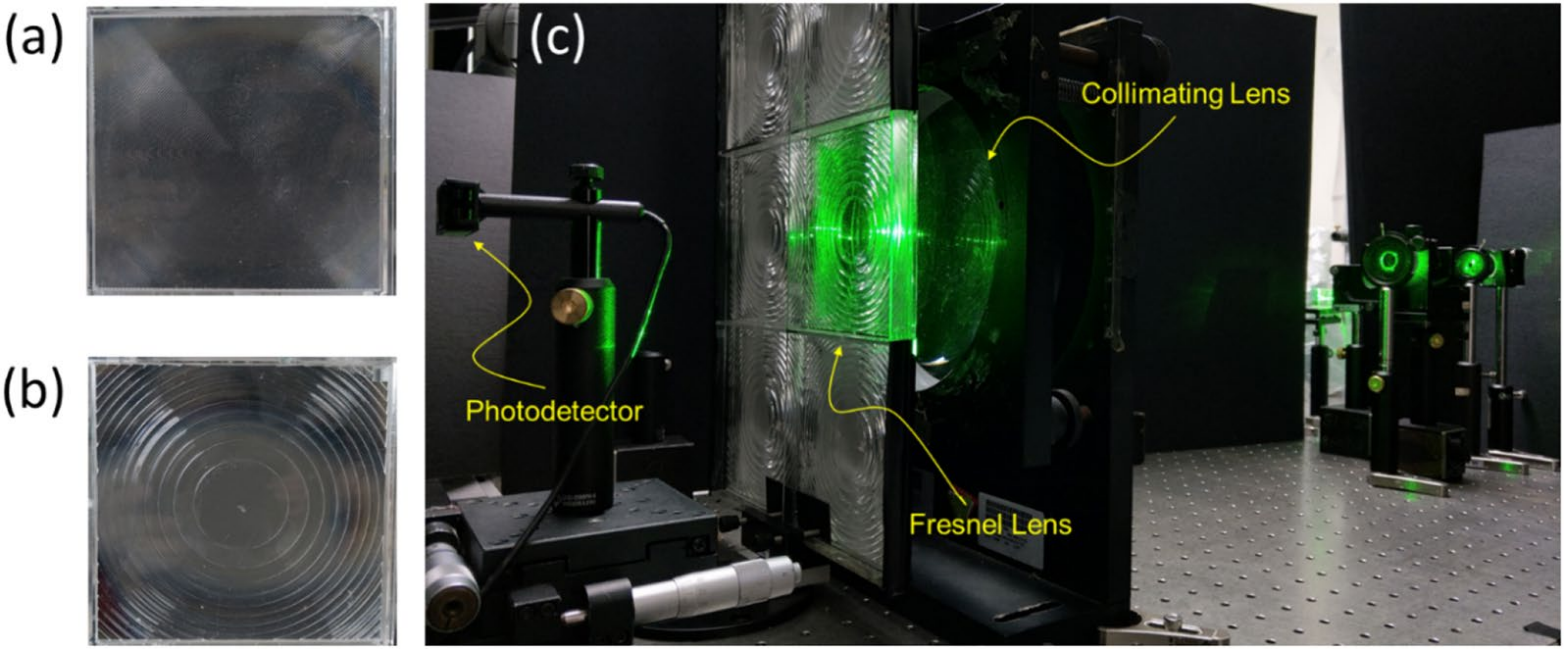
Light leakage measurements of the Fresnel lenses in the lab. (**a**) The thinner Fresnel lens (Fresnel lens #1): 60 main segments with a thickness of 0.56 mm. (**b**) The thicker Fresnel lens (Fresnel lens #2): 9 main segments with a thickness of 1.81 mm. Both two Fresnel lenses have the same radius. (**c**) The wavelength of light irradiation is 532 nm, and the power is around 100 mW. With the collimation lens, the light beam fully covers the Fresnel lenses.

As far as the structure of the Fresnel lens is concerned, neither the draft facet nor the RVRA caused by mold manufacturing can make the incident light converge on the same focal area. Therefore, a light leakage area is formed in the HCPV cell module. The draft facet is a side effect of the lens thinning process. The more the draft facet, the thinner the Fresnel lens. Another thing that occurs along with the draft facet is the round angle. The round angle is generated due to the large angle turning in the structure of the Fresnel lens. Similarly, the round angle structure cannot concentrate the sunlight in the central region. In the fabrication process of the Fresnel lens, a draft angle of at least 1° is required during the demolding process. The round angle usually has a radius of curvature of hundreds of microns or more. These factors can make the light concentration situation even worse. We can estimate its influence on light concentration according to the area ratio of the draft facet structure and the round angle in the Fresnel lens structure. The light leakage L caused by the draft facet and the round angle can be expressed

1 $${\text{L}} = \frac{{A_{G} + A_{R} }}{{A_{F} }} \times 100\% ,$$

where the A_F_ is the projection area of the Fresnel lens. A_G_ is the overall projection area of the draft facet, and A_R_ is the projection area occupied by the round angle. Therefore, when the Fresnel lens is thinner, the number of segments will increase, so the proportion of light leakage will also increase. To verify the above statement, we used two Fresnel lenses with similar areas for luminous flux measurement, as shown in Fig. 2. In the experiment, a monochromatic laser beam with a central wavelength of 532 nm was exploited to conduct light concentration experiments. As shown in Fig. 2c, the laser light passed through first an objective lens and then a large-aperture lens to form the collimated light beam. We used this collimated beam to mimic the characteristics of outdoor sunlight. The two Fresnel lenses are shown in Fig. 2, respectively, in the light irradiation path. Fresnel lens #1 is a thinner lens with more segments, and Fresnel lens #2 is a thicker lens with fewer segments. In the end, a light detector with a rectangle aperture of 10 mm × 16 mm was located at its focal plane to measure the luminous flux of the focusing spot. The light leakage ratios of the two lenses were measured 43% and 36%, respectively. Since the simulation shown in Fig. 1 was for the thicker Fresnel lens #2, we could compare the measurement result with the simulation shown in Fig. 1. The light leakage of the measurement was 36% when the focusing area was 10 mm × 16 mm. The simulation of the light leakage was 37% when the focusing area was set 1.1 mm × 1.1 mm, which was much smaller than that in the measurement. The difference in the focusing area can be explained as follows. Although the major important optical parameters of the Fresnel lens were set in the simulation, the simulated Fresnel lens was still in an ideal condition. It means that there was no manufacturing error, and the incident light was well-collimated. These two factors were not possible in the experiment. Thus the focusing spot in the experiment could be laterally extended or blurred in comparison with that in an ideal case, such as in the simulation. However, the simulation and the experimental measurement showed that around 36–37% light leakage could be observed outside the focusing area in both the simulation and the experiment. Without a doubt, the leakage mechanism of a Fresnel lens was well proved.

**Figure 3:**
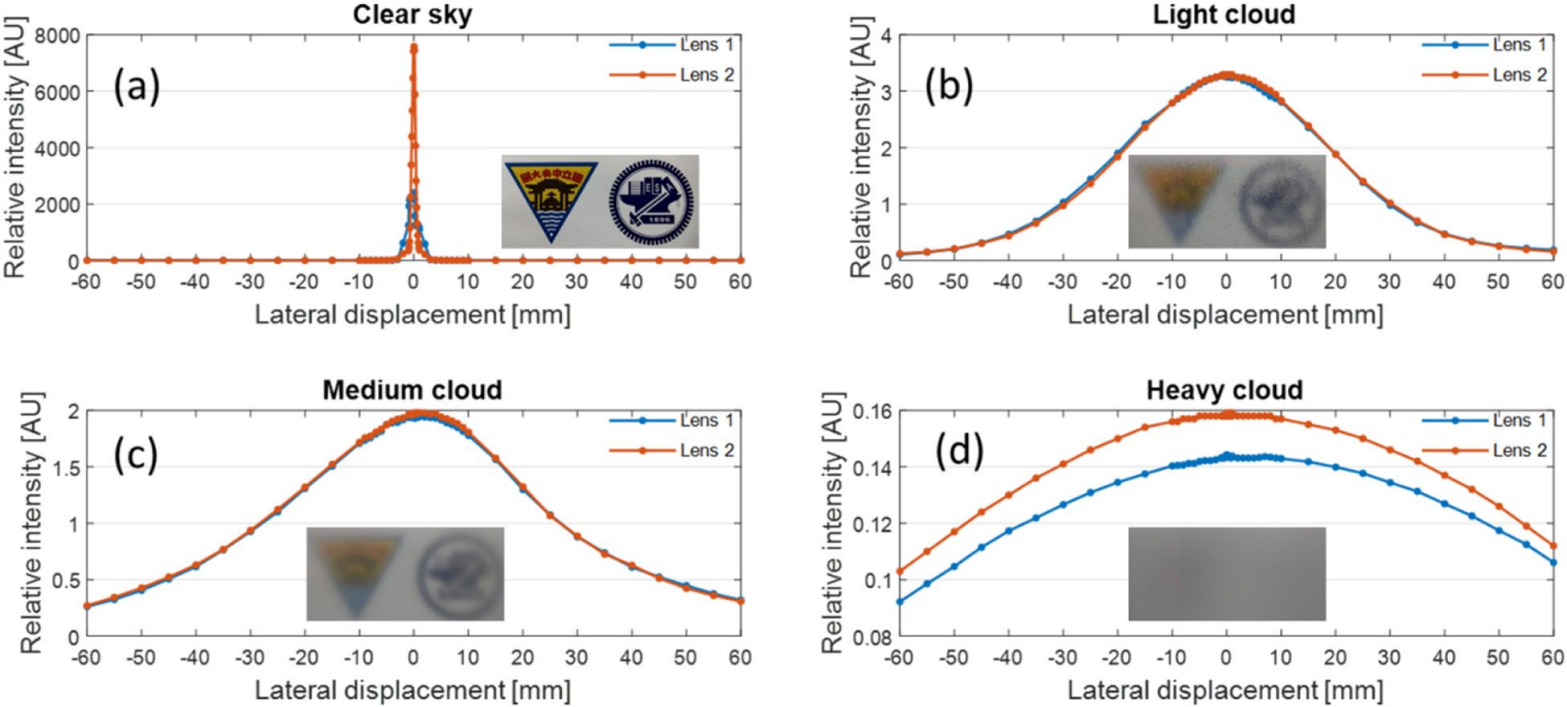
Lens focusing performance with the use of diffusive glasses to mimic weather at different conditions. The one-shot transmittances of the diffusers are (**a**) 100% without a diffuser for the clear sky, (**b**) 83% for the light cloud, (**c**) 65% for the medium cloud, and (**d**) 56% for the heavy cloud.

Another HCPV applicability problem results from wavefront disorder caused by clouds when sunlight passes through the earth’s atmosphere. When light waves pass through clouds with considerable thickness, part of the light will collide with water droplets, cause light refraction or reflection, and finally form random light scattering. Therefore, when the light enters a Fresnel lens, it can no longer be regarded as parallel light, while the concentrated beam will not focus on the center point of the HCPV module. To prove this effect, we used the experimental configuration presented in Fig. 2 and placed different diffusers between the collimating and Fresnel lenses. The three diffusers had different one-shot transmittances, defined as the ratio of the penetrating light flux and the incidence flux of a certain diffuser^20^. These diffusers allow different penetration ratios of the collimating beam, which could be used to simulate the influence of clouds on sunlight. The experiment results are summarized in Fig. 3. In Fig. 3a, a clear concentrated beam spot could be observed around the center of the detected area, indicating that most of the energy was collected at a limited location. Such a result might be used to mimic the days with a clear sky. From Fig. 3b–d, we could observe from the photos that the background patterns became increasingly blurred. At the same time, the intensity in the central area decreased, and the illuminance of the surrounding area was closer and closer to the intensity in the central area. Fresnel lenses #1(blue) and #2 (red) exhibited similar properties.

**Figure 4:**
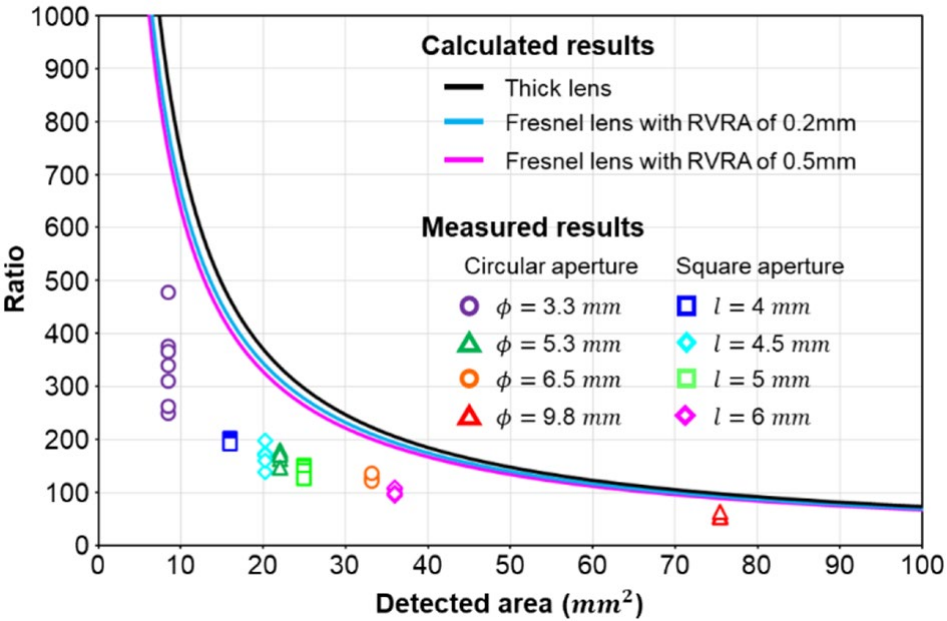
Fresnel lens performance measurements under sunlight. When the aperture size and shape were changed, the measurement was done under clear sunlight with Fresnel lens #1. The three curves show the related simulations.

The light source used in the laboratory was derived from a green laser diode, and it did not fit the practical condition. To understand the optical effect affected by the Fresnel lens's structure, we needed to compare the illuminance by the sunlight illumination and by the corresponding simulation. Therefore, we measured the illuminance of the focused sunlight with the Fresnel lens. In the measurements, we chose Fresnel lens #2 for experiments and used a photodetector (Thorlabs PM16-12) that can be utilized at high-power illumination to measure the power of the focal spot point. Since the measurement was done under moving sunlight, and the focusing spot was not an ideal tiny spot, the illuminance related to the aperture size of the photodetector. The result was that precise measurement of the focusing sunlight became difficult. Alternatively, we changed the aperture of the photodetector to collect more data and tried to find the correlation between the illuminance and the aperture size. The measurement results are shown in Fig. 4, where the vertical axis indicates the illuminance ratio at the focus spot and the ground without a focusing lens. There are three simulation curves. The black curve is the calculated focused illuminance ratio for a conventional lens with the same f-number. The blue and pink curves are obtained using Fresnel lenses with RVRA of 0.2 mm and 0.5 mm, respectively. The slight difference among the three curves was caused by the geometrical structures of the Fresnel lens. The situation of the measurement was different. The moving sunlight made the illuminance measurement through a tiny aperture difficult. Therefore, we decided to change the aperture size and shape (including circle and square). The measurement is shown in Fig. 4, where the measurement result shows a similar trend of the ratio as a function of the aperture area. Thus, the Fresnel lens simulation helped predict the optical property of sunlight.

## Field measurement and analysis

To understand the concentrating efficiency and light leakage characteristics of the HCPV module under actual weather conditions, we still selected Fresnel lens #2 for all the following field measurement experiments. We set up a 2 × 2 Fresnel lens array on a box with two rotation dimensions. The Fresnel lens array can be manually adjusted to face directly toward the sun at any time. During the measurement, different weather conditions occurred as the thickness of the cloud layer changed. Therefore, we could observe at the bottom of the box that the brightness of the light leakage area will vary depending on the change in the cloud thickness. The illuminance of this area was the physical quantity to be measured in this experiment.

**Figure 5:**
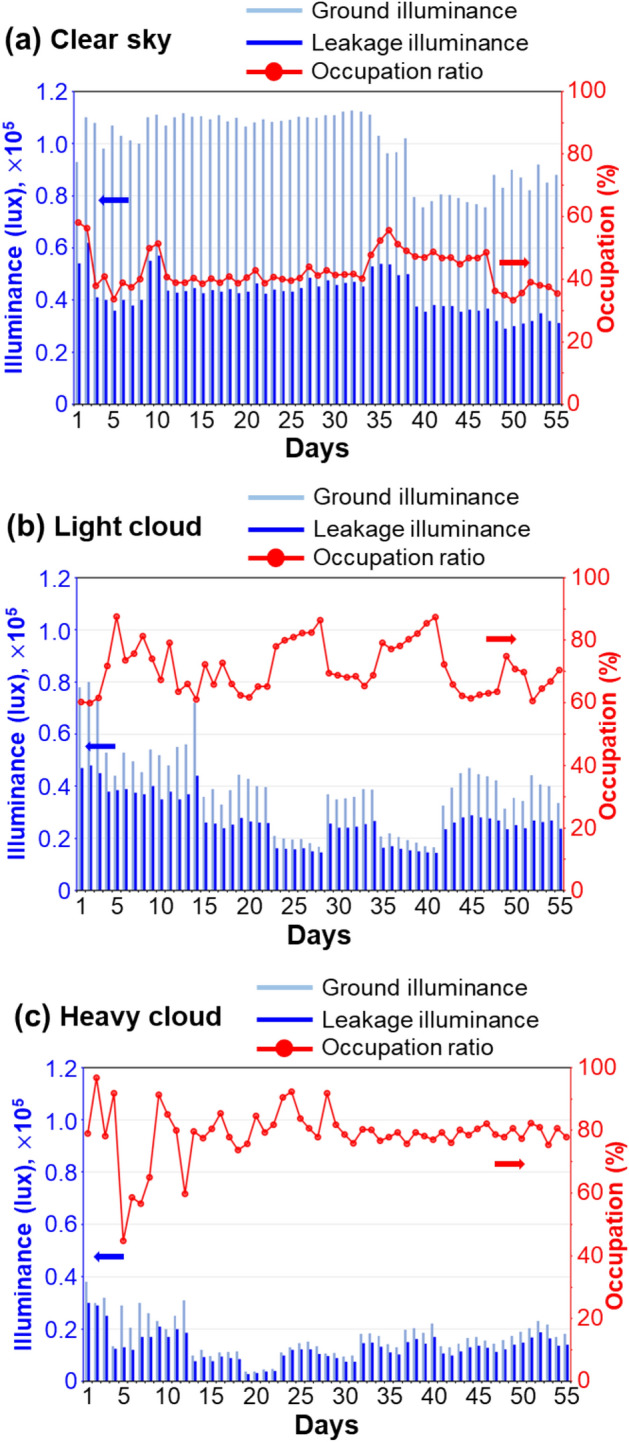
Field measurement results under different weather conditions. (**a**) Clear sky, (**b**) light cloud, and (**c**) heavy cloud. Even on cloudy days, the area where the light dissipates can still have nearly 80% of the light due to the presence of the lens. This is quite helpful for developing solar photovoltaic and agro-ecological balance at the same time.

During the half-year measurement process, we can roughly divide the weather conditions into three situations according to the thickness of the clouds. The first is a clear sky, that is, there are no clouds when looking at the sky, and the ground illuminance exceeds 100,000 lx. The second is a lightly cloudy sky, that is, the thickness of the cloud layer is thin, and the sun can still be vaguely seen through the cloud layer. On light cloudy days, part of the light is still parallel, so the focus point can still be clearly observed. However, the light intensity at the focal point has been greatly reduced compared with the clear sky. The third is a heavy cloudy sky, that is, the cloud layer is thicker, and the brightness of the whole sky is more uniform. In this kind of weather, it takes work to know the correct position of the sun. That is to say, after the clouds scatter the sunlight, its wavefront is irregularly distributed. At this time, no focus point can be seen at the bottom of the box. After more than six months of measurements, we measured the ground illumination and light leakage under different weather conditions. The measured results and the ratio between the two physical quantities are summarized in Fig. 5. Figure 5a–c shows the values of the ground illuminance (light-blue bars) and the leakage illuminance (dark-blue bars) for clear sky, lightly cloudy, and heavily cloudy days, respectively. The figure’s red curves record the occupation ratios of light intensity in the light leakage area under a clear sky, lightly cloudy, and heavily cloudy days, which were about 40%, 65%, and 80%, respectively. The experimental results show that the percentage of light leakage under the Fresnel lens was higher when the cloud layer was thicker. The ground illumination value decreased with the increase in cloud thickness, which means that the energy converged by the Fresnel lens at the focus point was also reduced.

From the above experimental results, it is important and interesting to find that the illuminance values of the light leakage area show no significant changes even at different cloud thicknesses. This is because the percentage of light leakage is lower when the ground illumination is higher. The ground illuminance value decreases when the cloud thickness increases, but the light leakage percentage rises. So we find that the illuminance of the leakage area will be close to a constant value regardless of the weather conditions. The light leakage of HCPV is caused by two optical phenomena, including the scattering of sunlight by clouds and the leakage of light caused by the structure of the Fresnel lens. This kind of light leakage should be adequately utilized. The first idea is to change the base plate of the conventional HCPV from a metal heat sink to a transparent medium so that light from outside the center can reach the ground. This distribution of sunlight between 20,000 and 40,000 lx should support the growth of some plants on the ground. Therefore, medium-illuminated agricultural activities can be carried out by switching to transparent baseboards. In addition, from a power generation point of view, we can also use low-cost solar panels to lay in the leakage area for power generation, so we designed a power generation installation that combines the advantages of both HCPV and polycrystalline solar panels.

## Hybrid photovoltaic device

**Figure 6:**
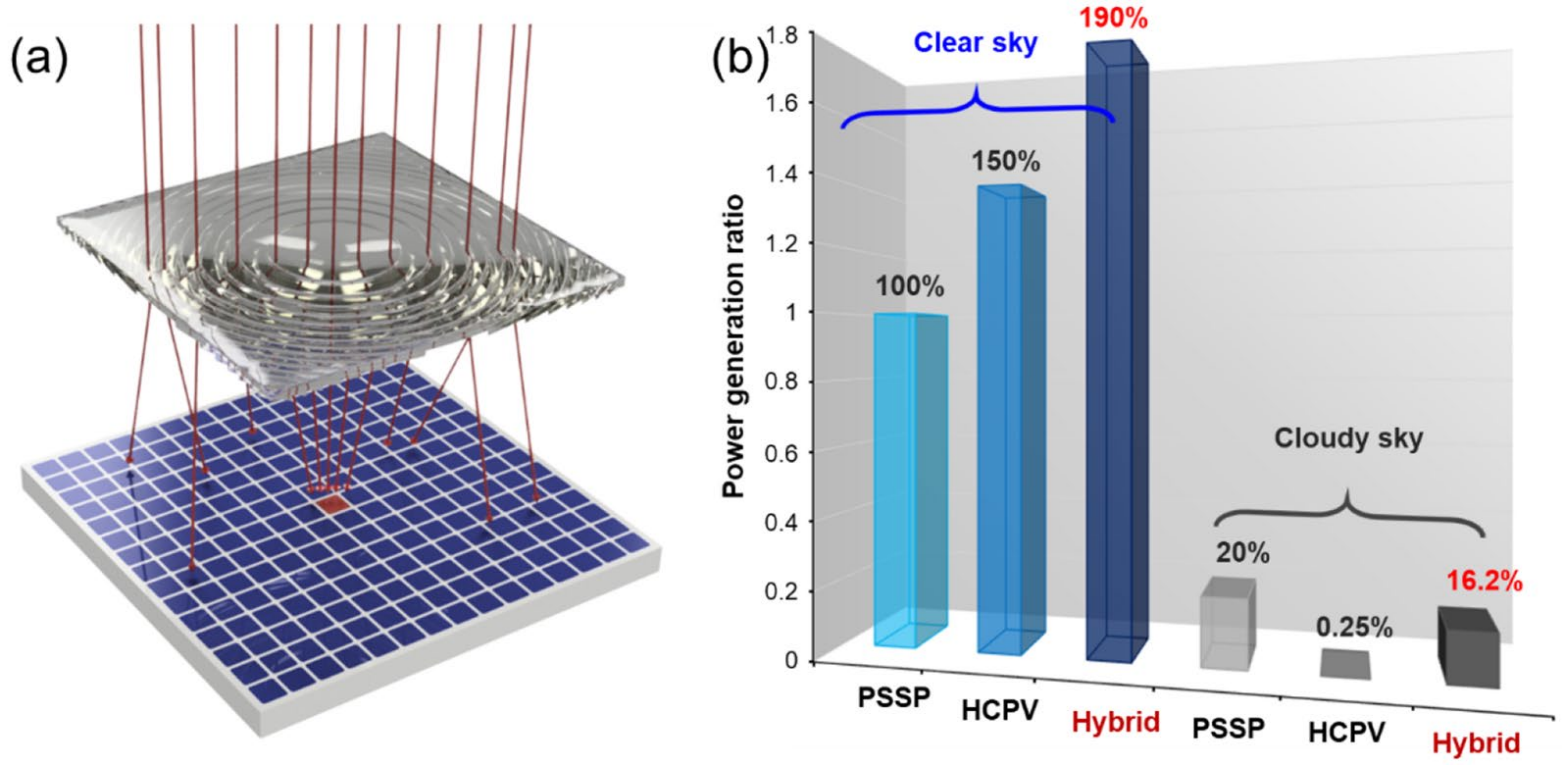
Proposed hybrid high-concentration photovoltaic device. (**a**) The schematic diagram of the proposed hybrid photovoltaic device includes a Fresnel lens, a high-quality III–V photovoltaic component located at the focused area, and conventional PSSP’s arranged around. (**b**) The comparisons of the power generation ratio among the three designs, where blue bars are for clear sky scenario and grey bars are for the heavy-cloud sky scenario referred to Fig. 5c.

A hybrid high-concentration photovoltaic system is designed and proposed by placing a high-efficiency III-V solar panel at the focus point and laying a polycrystalline silicon-based solar panel around it, as schematically shown in Fig. 6a. In the schematic diagram in Fig. 6a, the parallel beam from the sun passes through the Fresnel lens and is focused on the high-efficiency solar panel. The light leakage from the Fresnel lens structure and the scattered light from the sunlight passing through the clouds can be directed to the polycrystalline silicon-based solar panel (PSSP) for power generation. When the sky is clear, the light is concentrated on the high-efficiency solar cell, so the power generation efficiency is high. In a heavy cloud, the sunlight cannot be concentrated on the high-efficiency solar cell, so a conventional HCPV cannot effectively generate electricity, but this design can still generate electricity by polycrystalline solar panels.

To estimate the power generation efficiency of our proposed hybrid high-concentration photovoltaic system under different weather conditions, we compared the power generation capacity of the conventional, high-concentration photovoltaic (HCPV) and hybrid HCPV power generation systems. First, we assume that the Fresnel lens area is about 163.8 cm^2^, while the area of the III–V solar panel is 0.75 cm^2^ so the area of the polycrystalline silicon solar panel is 163.05 cm^2^. Since the photon conversion efficiencies are 15% and 45% for the PSSP and III-V compound semiconductors, we assume conversion efficiencies of α and 3α for a PSSP and an III-V solar panel, respectively. Based on the above assumption, we will calculate power generation for different solar cells under various cloud conditions.

The first is the clear sky scenario referred to in the conditions of Fig. 5a. For simplicity, we assume that the ground illumination is 100,000 lx. Therefore, the power generation of a PSSP in a clear sky can be calculated

2 $$P_{PSSP} = 163.8 \times {\upalpha } \times 100,000$$

The measurement shown in Fig. 5a indicates that the light leakage under the Fresnel lens is 40% of the ground illuminance without the Fresnel lens. We assume that the optical flux at the focusing area of the HCPV is the rest power transmitted through the Fresnel lens where there is an additional 10% of Fresnel loss. The power generation of the HCPV (P_*HCPV*_) and the hybrid HCPV (P_*hybrid*_) can be respectively written

3 $${\text{P}}_{HCPV} = \left( {1 - 0.1 - 0.4} \right) \times 3 \times P_{PSSP} = 1.5P_{PSSP}$$

4 $${\text{P}}_{hybrid} = {\text{P}}_{HCPV} + 40\% \times P_{PSSP} = 1.9P_{PSSP}$$

Equations (3) and (4) indicate that the hybrid HCPV could reserve 40% power leakage under the sunlight in a clear sky, and finally reaches around twice the power generation of a PSSP, as shown in the blue bars in Fig. 6b.

For the next, we discuss the case of heavy-cloud days referred to the conditions of Fig. 5c. Here, we assume that the illuminance on the ground is 20% of that under the clear sky, i.e., 20,000 lx or 0.2P_PSSP_. Therefore, the power generation of the PSSP is about 0.2 P_PSSP_. From Fig. 5c, the light leakage of the Fresnel lens is set at 80%, so the power generation in the light leakage area is around 0.16 P_PSSP_. In such conditions, there is no prominent focusing spot at the tightened III-V solar cell, as shown in Fig. 3d, so the power generation of the HCPV can be calculated around P_PSSP_/400. It means that the HCPV suffers from the heavy cloud in the sky and has no function under such a condition. However, the hybrid HCPV can still reserve 80% of the sunlight owing to light leakage, and the total power generation is around 0.16 P_PSSP_, as shown in the grey bars in Fig. 6b. As a result, the hybrid HCPV acts better than an HCPV in a clear sky and like a PSSP in a heavy-cloud sky.

The comparison of the power generation ratio of different solar photovoltaic devices is summarized, as shown in Fig. 6b. The calculation results show that a hybrid HCPV generates more electricity than an HCPV in all scenarios by collecting the leakage light of the Fresnel lens, and power generation is close to that of a PSSP on heavy cloudy days when an HCPV losses its function. The proposed hybrid HCPV is a new design that can improve power generation efficiency and adapt to various cloud conditions.

## Conclusion

In this paper, we started with the theoretical and practical studies of a Fresnel lens and analyzed the inherent light leakage characteristic when using a Fresnel lens as a focusing lens. We pointed out that the light leakage is also caused by cloud scattering, which is an unavoidable factor for an HCPV. Then a series of experiments were done with the corresponding calculations to prove the light leakage mechanism, including from the Fresnel lens and the clouds. The corresponding field measurement with the use of a Fresnel lens to focus the sunlight was done for six months for various cloud sky conditions.

According to the acquired results, the most valuable finding was that the solar concentrator based on the Fresnel lens has an illuminance of about 20,000–40,000 lx in the light leakage region, whether it is a sunny day or a cloudy day with different cloud thicknesses. This is an inherent characteristic of HCPV based on the Fresnel lens and the national sunlight characteristics. Such a property could be helpful in solar power factories with agricultural benefits.

This finding prompted us to propose a hybrid high-concentration solar module in which more cost-effective PSSPs are placed around the high-efficiency wafer of HCPV to capture the dissipated light leakage and convert it into usable electricity. Such a system may exhibit extremely high conversion efficiency under different cloud conditions. Under the assumption that the conversion efficiencies are 15% and 45% of the PSSP and III–V compound semiconductors, respectively, the proposed hybrid system could reach 190% and 126% power generation efficiency compared with PSSP and HCPV systems, respectively, in the clear sky. When there are heavy clouds, the HCPV system has almost no power generation efficiency, while the proposed hybrid system can maintain more than 80% of the power generation efficiency of the PSSP system. Thus the proposed novel solar power system is useful for reaching optimal solar power generation under different cloudy skies.

## Data Availability

All datasets from this study are available from the corresponding author upon reasonable request.
